# Supplementary material for: Comparative multiomics analysis of cell physiological state after culture in a basket bioreactor
Source: Sci Rep. 2022 Nov 23;12:20161. doi: 10.1038/s41598-022-24687-4 (PMC9686226; doi:10.1038/s41598-022-24687-4)
Supplement: Supplementary file 1 — Supplementary Information 1. [file 41598_2022_24687_MOESM1_ESM.zip › raw data/Proteomic raw data/6.KEGGenrichment/EG.vs.CG/DOWN/pathway/src/04151.html]

map04151


- K00693

- Down regulated proteins

A0A0D9R6H1(Description:Glycogen [starch] synthase;Gene:GYS2;log2FC:-1.1230)

A0A0D9S336(Description:Glycogen [starch] synthase;Gene:GYS1;log2FC:-1.6625)

- K04357, K08774, K18496, K18497, K04358, K05451, K02582, K04355, K04356, K12457, K04526, K05459, K13769, K04359, K17386, K05450, K05453, K05461, K05454, K05448, K16858, K16859, K05449, K05460, K05465, K05466, K05467, K05462

- Down regulated proteins


A0A0D9S6I0(Description:Macrophage colony-stimulating factor 1;Gene:CSF1;log2FC:-1.2197)

- K03900, K16857, K04659, K05635, K05636, K05637, K05717, K06236, K19719, K06237, K06238, K08131, K06240, K06241, K06243, K06244, K06245, K06246, K06247, K06248, K06249, K06250, K06251, K06252, K06253

- Down regulated proteins


A0A0D9R9X4(Description:Fibronectin;Gene:FN1;log2FC:-1.1754)

A0A0D9R486(Description:Collagen type IV alpha 3 chain;Gene:COL4A3;log2FC:-1.4852)

A0A0D9RY11(Description:Collagen type IV alpha 2 chain;Gene:COL4A2;log2FC:-1.7446)

A0A0D9RY12(Description:Collagen IV NC1 domain-containing protein;Gene:COL4A1;log2FC:-1.2021)

Close
